# Early Success With Retention in Care Among People Living With HIV at Decentralized ART Satellite Sites in Yangon, Myanmar, 2015–2016

**DOI:** 10.3389/fpubh.2019.00124

**Published:** 2019-05-22

**Authors:** Kyaw Myo Htet, Kyaw Thu Soe, Myo Minn Oo, San Hone, Suman S. Majumdar, Htun Nyunt Oo

**Affiliations:** ^1^National AIDS Program, Department of Public Health, Ministry of Health and Sports, Naypyitaw, Myanmar; ^2^Department of Medical Research (Pyin Oo Lwin Branch), Mandalay, Myanmar; ^3^The International Union Against Tuberculosis and Lung Disease, Mandalay, Myanmar; ^4^International Development, Burnet Institute, Melbourne, VIC, Australia

**Keywords:** retension, decentralized satellite sites, Myanmar (Burma), operational research, HIV

## Abstract

**Introduction:** Myanmar is one of the countries in the Asia-Pacific region hit hardest by the HIV epidemic that is concentrated among urban areas and key populations. In 2014, the National AIDS Programme (NAP) launched a new model of decentralized service delivery with the establishment ART satellite sites with care delivered by HIV peer workers.

**Methods:** ART satellite sites are implemented by non-government organizations to service high burden HIV areas and populations that suffer stigma or find access to public sector services difficult. They provide continuity of HIV care from outreach testing, counseling, linkage to care, and retention in care. Anti-retroviral (ART) initiation occurs at health facilities by specialist physicians. We conducted a retrospective cohort study of people living with HIV (PLHIV) who were initiated on ART from 2015 to 2016 at five ART satellite sites in Yangon, Myanmar to assess outcomes and time from enrolment to ART initiation.

**Results:** Of 1,339 PLHIV on ART treatment in 2015–16, 1,157 (89%) were retained, and 5% were lost from care and 5% reported dead, at the end of March 2018. Attrition rates (death and lost-to-follow-up) were found to be significantly associated with a CD4 count ≤ 50 cells/mm^3^ and having baseline weight ≤ 50 kg. Median time taken from enrolment to ART initiation was 1.9 months (interquartile range: 1.4–2.5).

**Conclusion:** We report high rates of retention in care of PLHIV in a new model of ART satellite sties in Yangon, Myanmar after 3 years of follow-up. The delays identified in time taken from enrolment to ART initiation need to be explored further and addressed. This initial study supports continuation of plans to scale-up ART satellite sites in Myanmar. To optimize outcomes for patients and the program and accelerate progress to reduce HIV transmission and end the HIV epidemic, operational research needs to be embedded within the response.

## Introduction

Globally, 36.7 million people were living with HIV/AIDS (PLHIV) in 2016 (1). More than 13% of these are from the Asia and Pacific region where the epidemic is concentrated in vulnerable groups or key populations (KPs)—people who inject drugs (PWID), men who have sex with men (MSM), and female sex workers (FSW) (2). Anti-retroviral therapy (ART) scale-up across the globe has contributed to a 48% decline in deaths from AIDS-related causes, which have peaked at 1.9 million in 2005 to 1 million in 2016 (1). The United Nationals Goal to end the HIV epidemic by 2030 will not be achieved unless access to treatment and care is provided to all, with a focus on key populations.

Myanmar is one of the countries in the region hit hardest by the HIV epidemic and is one of the 35 UNAIDS fast track priority countries that account for 90% of new HIV infections globally. The Myanmar Ministry of Health and Sports recognizes HIV as a priority public health issue and implements the response through the National AIDS Program (NAP) in collaboration with national and international non-governmental organizations (iNGOs). The Myanmar National Strategic Plan (NSP III) on HIV and AIDS (2016–2020) aims to end HIV as a public health threat in Myanmar by 2030 through fast tracking access to continuum of integrated and high quality services that protect and promote human rights for all (3).

Myanmar has a concentrated HIV epidemic among urban areas and key populations (4). There has been good progress toward reducing the burden of disease evidence by a decline in prevalence among the general adult population from 0.9% in 2000 to 0.6% in 2016 (4). A significant decline also has been seen in HIV-related mortality (3). Despite this progress, challenges remain: a large proportion of the estimated PLHIV do not know their HIV status and approximately half are still not receiving life-sustaining antiretroviral therapy (ART) (3). Access to services, stigma and discrimination remain substantial barriers to improving outcomes. The current NSP utilizes several new strategies including different service delivery approaches such as geographic prioritization, reaching priority populations and partnerships between the public, NGO, community, and private sectors.

## Background and Rationale

In Myanmar HIV treatment and care services were initially piloted and scaled up by iNGOs from 2002. The NAP commenced ART care provision in 2005. The public sector aims to support ~75% of total national cohort by 2020. The 2013 World Health Organization (WHO) consolidated ART guidelines recommended HIV service integration and decentralization of HIV care based on systematic reviews that showed decentralization improves patient access to and retention in care (5, 6).

In 2012, NAP has commenced the ART decentralized (DC) sites in order to reduce the workload of ART centers and improve access to care for patients. Key populations suffer from stigma and discrimination and may find access to public sector ART centers difficult.

The national estimates of Population Size Estimates for KPs was 83,000 for PWID in 2014, and 66,000, and 255,000 for FSW and MSM, respectively, in 2016. The prevalence of HIV in MSM in Yangon is the highest in the Asia-Pacific region from a single location (3).

In 2014, the NAP launched a new service delivery model to reach urban PLHIV and key populations through clinic-based testing and treatment sites in partnership with iNGOs called ART satellite sites. Several studies have reported retention in care with rates ranging from (30 to 90%) (7–11). There have been no previous studies assessing the outcomes of PLHIV on ART at ART satellite sites in Myanmar. As the country is fast-tracking its HIV response under the new national plan, we report on a cohort of PLHIV under care at five ART satellite sites in Yangon from 2015 to 2016 and assess the programmatic outcomes of this model. The specific objectives are to UNAIDS (1) describe their demographic and clinical characteristics (2) determine retention in care over the period of January 2015 to March 2018 and (3) assess time from enrolment to ART initiation, and (4) determine factors associated with attrition defined as death or lost to follow-up (LTFU).

## Methods

### Essential Elements of the Intervention

Myanmar, population 51,486,253, is divided administratively, into Nay Pyi Taw Council Territory and 14 States and Regions with 330 townships (12). The NAP ART services are currently delivered through 127 ART centers, and 174 decentralized sites which may be at the township level, sub-township level and station health units or rural health center, as indicated by burden of disease (3). HIV testing services (HTS) are provided in more than 300 townships (13). Voluntary Confidential Counseling and Testing (VCCT) for HIV in Myanmar is available at all ART centers, decentralized ART sites, government hospitals and through NGOs. All ART centers under the NAP provide free HIV treatment and care with the support from the Global Fund against AIDS, TB, and Malaria (GFATM) in addition to the Government's contribution. On diagnosis with HIV, the individual is referred to the ART center, enrolled in the pre-ART register and clinical assessment and baseline investigations are performed. On ART initiation the individual is enrolled in the standardized NAP ART register and an individualized treatment card is commenced. Details of NAP treatment guidelines in Myanmar have been described previously (14).

In 2014, NAP launched the establishment of first decentralized ART centers or “satellite sites” to increase access to HIV care and services through a community-based peer worker model. The ART satellite site is responsible for on-going HIV care including follow-up visits (monthly initial visits after enrolment up to 6 months, followed by 3 monthly visits thereafter), monitoring, nutritional support, side-effect, and provision of opportunistic infection prophylaxis for PLHIV enrolled into that site. Decentralized ART satellite sites were established in townships based on three criteria: (1) high HIV burden (2) populations served by the site experience stigma and difficulty in accessing public sector ART centers (e.g., key population, migrant/mobile population) or (3) are an area in which well-established community-based implementing partner exists. As of December, 2017, 28 such centers had been established across 10 states and regions, all by iNGO partners and are under the supervision of NAP Myanmar. The satellite sites are staffed by peer workers (10 to 20 per site) who are supervised by a medical doctor. Peer workers perform community-based outreach to key populations, conduct HIV counseling and refer people to the ART satellite sites for rapid HIV testing. If the result of a person is HIV reactive, they are accompanied to the NAP ART centers for confirmation, general assessment, further counseling, and initiation of ART.

### Study Design and Population

We conducted a retrospective cohort study of PLHIV who were initiated on ART from 2015 to 2016 at five ART satellite sites in Yangon, Myanmar (Annex 1).

Sampling design and recruitment of subjects: We purposively selected all the ART satellite sites in Myanmar. We recruited the sites from one organization that gave consent and collaborated to participate in this study. All patients belonged to recruited sites were involved in this study.

Sample size calculation: It is not relevant in this study.

Fidelity of the practice of model: Each organization which implemented ART satellite sites models had to follow standard guidelines by NAP to reduce the variations among different sites. Monitoring and supervision was done regularly by central NAP team.

### Definitions of Outcomes

Each patient initiated on ART was assigned one of these four mutually exclusive outcomes as on 31th March, 2018 (date of censoring).

Retained in care: Patients who are alive and continue to receive ART care.

Death: death from any cause notified by medical staff or reported by family or next of kin.

Lost to follow up (LTFU) –defined as not attending for follow-up visits within 3 months after last scheduled date.

Transfer out: patients who were transferred to other clinics for continuation of care.

### Data Variables, Sources of Data, and Data Collection

The source of data was the electronic database of the iNGO which implemented decentralized ART satellite sites. The demographic and clinical variables included in the study were date of enrolment, age, sex, marital status, residence, risk group, entry, baseline WHO staging, weight in kg, CD4 count, date of ART initiation, type of ART regimen, programmatic outcome, and date of outcome. Data for these variables were cleaned and exported for data analysis. Patients' records were anonymized and de-identified prior to the analysis.

### Analysis and Statistics

Data were extracted from the electronic databased, reviewed extensively, and validated in STATA version 14.0 (College Station, Texas 77845 USA) prior to analysis. Baseline demographic and clinical characteristics were summarized using frequency and proportions. The proportions of death and LTFU cases were tabulated across baseline characteristics. Attrition was calculated by dividing the number of those LTFU or dead with the study population. The risk time started at the time of ART initiation. The date of LTFU or death was the date of failure event and for those who were retained, the date of last visit was the censored date. Those who were transferred out were also regarded as being retained under the NAP and hence date of the transfer was also censored. Overall and strata-specific attrition rates were also estimated per 100 person-years with 95% confidence interval and associated baseline factors compared using unadjusted hazard ratios (HR). Proportionality assumptions were tested using Schoenfeld residuals and log-log plots. A multivariate Cox proportional hazard regression model where the bivariate analysis showed *P*-value < 0.2 was used to adjust for confounders. *P*-values < 0.05 were regarded as significant.

### Ethics

Ethics approval was obtained from Ethics Review Committee of Department of Medical Research, Ministry of Health and Sports, Myanmar (ERC Approval Number Ethics/DMR/2018/036) and the Ethics Advisory Group of The Union, Paris, France.

## Results

**Figure 1:**
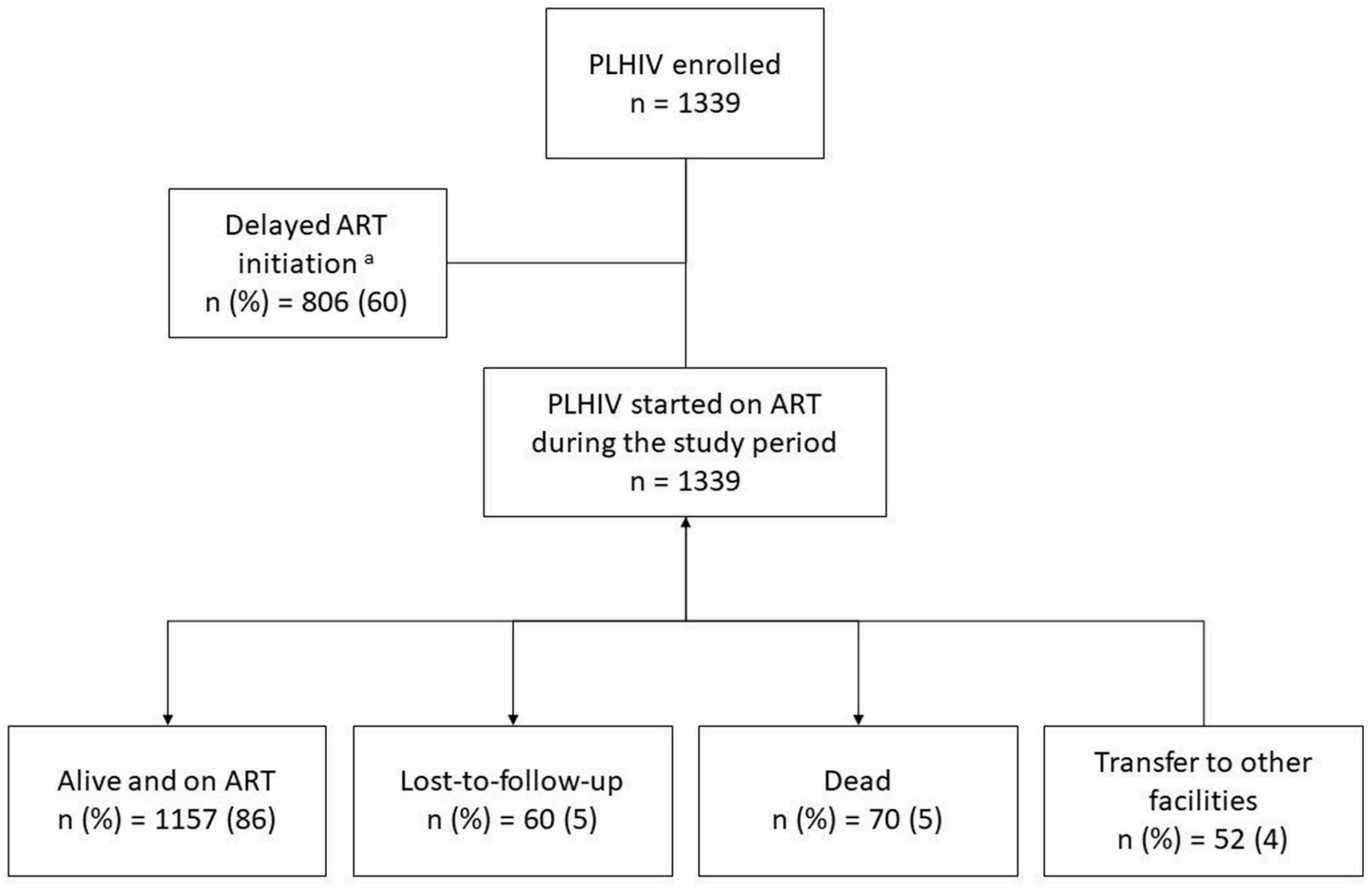
The programmatic outcomes in PLHIV initiated on ART between 2015 and 2016 at selected ART satellite sites, Yangon, Myanmar. PLHIV, people living with HIV; ART, anti-retroviral therapy.

In this study, a total of 1,339 PLHIV were initiated on ART between 2015 and 2016 at ART satellite sites. Among them, 1,157 (89%) were retained at the end of study period (March 2018), 5% were lost from care, and 5% reported dead (see Figure 1). Their clinical and demographic characteristics are displayed in Table 1. The predominant characteristics were being male, aged 31–40 years, heterosexual, married, and on ART initiation having WHO stage 3 or 4, weight ≤ 43 Kg, and baseline CD4 cells count ≤ 350 cells/mm^3^. Compared to LTFU, the proportions of dead were higher among those with widowed or separated marital status, WHO staging 3 or 4, decreasing levels of CD4 count ( ≤ 200 cells/mm^3^) and non-TDF based ART regimen. Date of enrolment was also retrospectively assessed in this treatment cohort and median time taken from enrolment to ART initiation was 1.9 months (interquartile range: 1.4–2.5).

**Table 1:** Demographic and clinical characteristics among PLHIV initiated on ART between 2015 and 2016 at ART satellite sites, Myanmar, disaggregated by LTFU, and death.

**Patient's characteristics**		Total *n* (%)^*^	Dead *n* (%)^*^	LTFU *n* (%)^*^
Total^**^		1,339 (100)	70 (5.2)	60 (4.5)
Age at ART initiation (years)	≤20	17 (1.3)	(0)	1 (1.7)
	21–30	389 (29.1)	21 (30)	34 (56.7)
	31–40	574 (42.9)	29 (41.4)	15 (25)
	>40	359 (26.8)	20 (28.6)	10 (16.7)
Sex	Male	714 (53.3)	48 (68.6)	30 (50)
	Female	625 (46.7)	22 (31.4)	30 (50)
Residence	Yangon	925 (69.1)	49 (70)	44 (73.3)
	Hlaing Thar Yar	284 (21.2)	16 (22.9)	16 (26.7)
	Others	127 (9.5)	4 (5.7)	(0)
	Not Recorded	3 (0.2)	1 (1.4)	(0)
Entry	MAM	789 (58.9)	45 (64.3)	39 (65)
	NGO	133 (9.9)	5 (7.1)	5 (8.3)
	Others	417 (31.1)	20 (28.6)	16 (26.7)
Marital status	Single	209 (15.6)	12 (17.1)	7 (11.7)
	Married	902 (67.4)	39 (55.7)	39 (65)
	Widowed	97 (7.2)	10 (14.3)	3 (5)
	Separated	131 (9.8)	9 (12.9)	11 (18.3)
Weight at ART initiation (Kg)^a^	<50	668 (49.9)	53 (75.7)	36 (60)
	≥50	671 (50.1)	17 (24.3)	24 (40)
CD4 count at ART initiation^a^	≤200	593 (44.3)	53 (75.7)	27 (45)
	201–350	271 (20.2)	3 (4.3)	11 (18.3)
	351–500	227 (17)	4 (5.7)	9 (15)
	>500	153 (11.4)	3 (4.3)	8 (13.3)
	Not recorded	95 (7.1)	7 (10)	5 (8.3)
ART regimen	TDF-based	1,294 (96.6)	64 (91.4)	59 (98.3)
	Others	45 (3.4)	6 (8.6)	1 (1.7)

*PLHIV, people living with HIV; ART, anti-retroviral therapy; Kg, kilogram; TDF, tenofovir; LTFU, lost-to-follow-up*.

* Column percentage;

** *row percentage*.

a *These variables were measured at the time of ART initiation*.

**Figure 2:**
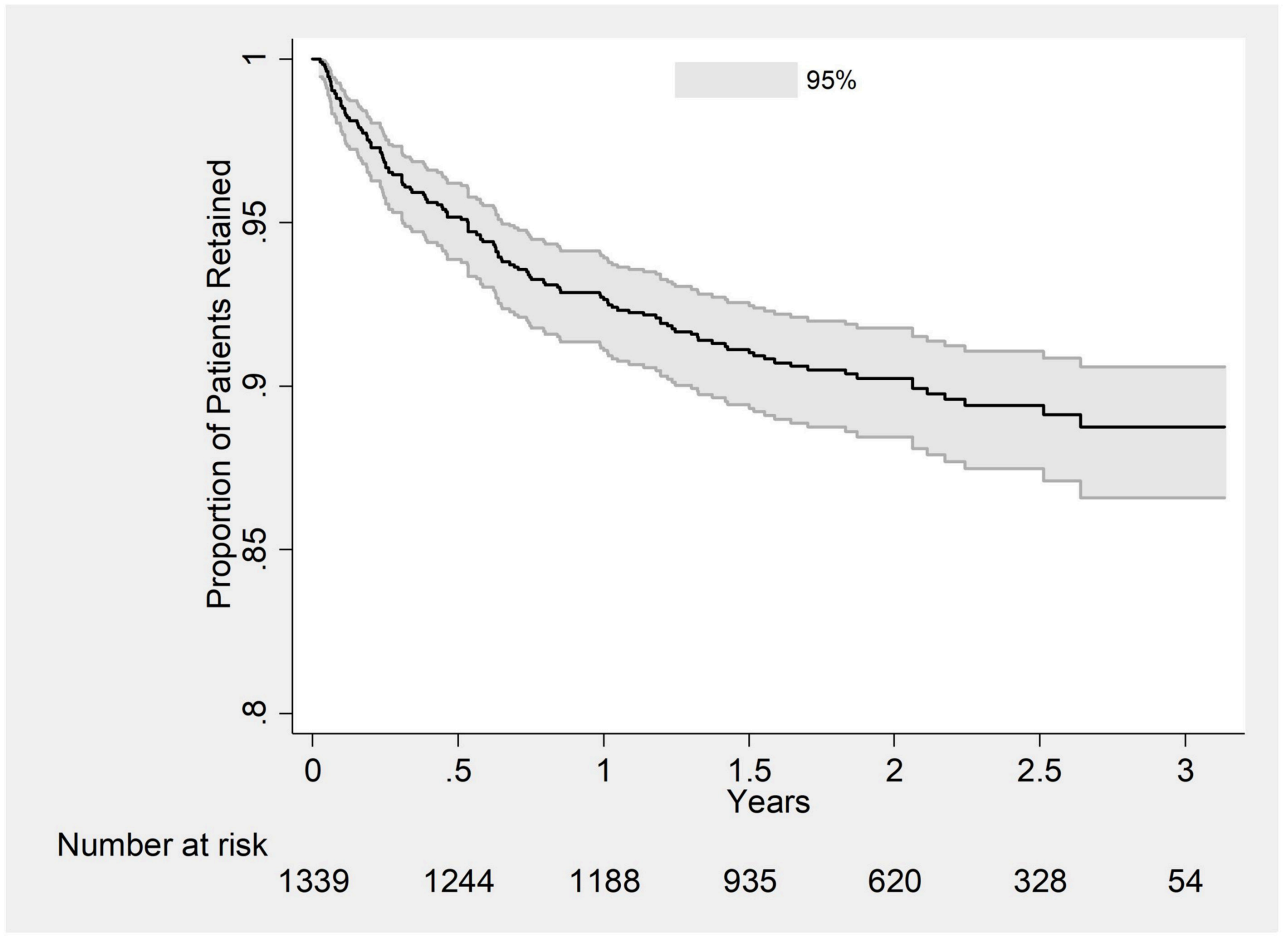
Kaplan-Meier Plot estimating retention in care in PLHIV initiated on ART between 2015 and 2016 at ART satellite sites, Myanmar.

A total of 2,502 person-year of follow-up was observed with a retention rate of 89% at 36 months (95%CI: 87–91) (see Figure 2). Attrition rates (dead and lost-to-follow-up) were particularly higher among those aged ≤ 30 years, who were widowed or separated, WHO staging 3 or 4, baseline weight ≤ 50 kg and CD4 cells count ≤ 50 cells/mm^3^ (see Table 2). Cumulative incidence of attrition was 12% (95% CI: 10–14) with 60% of all cases happening during the first year on ART (see Table 3). Age group ≤ 30, weight ≤ 50 kg and baseline CD4 count ≤ 50 cells/mm^3^ were independent risk factors for attrition.

**Table 2:** Attrition (LTFU and Dead) and associated factors among PLHIV initiated on ART between 2015 and 2016 at ART satellite sites, Myanmar.

**Patient's characteristics**		**Total *n* (%)**	**Attrition** ***n*** (%)^*^		**Attrition rate per 100 Pys (95% CI)**	**uHR (95% CI)**	**aHR (95% CI)**
Total		1,339 (100)	130	(10)	5.2 (4.4–6.2)		
Age at ART initiation (years)	≤30	406 (30)	56	(14)	7.7 (5.9–10)	**1.9 (1.3–2.8)**	**1.8 (1.2–2.8)**
	31–40	574 (43)	44	(8)	4 (3–5.4)	1	1
	>40	359 (27)	30	(8)	4.4 (3.1–6.3)	1.1 (0.7–1.7)	0.9 (0.5–1.5)
Sex	Male	714 (53)	78	(11)	5.9 (4.7–7.3)	1.3 (0.9–1.9)	1.5 (1–2.3)
	Female	625 (47)	52	(8)	4.4 (3.4–5.8)	1	1
Residence	Yangon	925 (69)	93	(10)	5.4 (4.4–6.7)	1	1
	HlaingTharYar	284 (21)	32	(11)	5.7 (4–8.1)	1.1 (0.7–1.6)	1.2 (0.8–1.8)
	Others	127 (9)	4	(3)	1.7 (0.7–4.6)	0.3 (0.1–0.9)	0.4 (0.1–1)
	Unknown	3 (< 1)	1	(33)	33.5 (4.7–240)	–	
Entry	MAM	789 (59)	84	(11)	5.7 (4.6–7.1)	1.4 (0.7–2.8)	–
	NGO	133 (10)	10	(8)	4 (2.1–7.4)	1	–
	Others	417 (31)	36	(9)	4.6 (3.3–6.3)	1.1 (0.6–2.3)	–
HIV transmission risk	Heterosexual	1,113 (83)	105	(9)	5 (4.1–6)	1	–
	MSM	36 (3)	1	(3)	1.4 (0.2–10)	0.3 (0–2)	–
	Sex Worker	75 (6)	7	(9)	5.2 (2.5–11)	1 (0.5–2.2)	–
	PWID	13 (1)	1	(8)	3.8 (0.5–27.1)	0.8 (0.1–5.6)	–
	Blood Transfusion	10 (1)	0	(0)	–	–	–
	MTCT	9 (1)	3	(33)	18.6 (6–57.6)	**3.5 (1.1–11.1)**	–
	Unknown	83 (6)	13	(16)	9.5 (5.5–16.3)	–	
Marital status	Single	209 (16)	19	(9)	4.7 (3–7.4)	1 (0.6–1.7)	0.7 (0.4–1.3)
	Married	902 (67)	78	(9)	4.6 (3.7–5.7)	1	1
	Widowed	97 (7)	13	(13)	7.2 (4.2–12.5)	1.6 (0.9–2.8)	1.9 (1–3.7)
	Separated	131 (10)	20	(15)	8.9 (5.7–13.7)	**1.9 (1.1–3.1)**	1.5 (0.9–2.6)
WHO staging^a^	1	422 (32)	23	(5)	2.9 (2–4.4)	1	1
	2	278 (21)	17	(6)	3.2 (2–5.2)	1.1 (0.6–2.1)	0.8 (0.4–1.7)
	3	533 (40)	74	(14)	7.7 (6.1–9.7)	**2.7 (1.7–4.2)**	1.7 (1–3.1)
	4	106 (8)	16	(15)	7 (4.3–11.4)	**2.7 (1.4–5.1)**	1.6 (0.7–3.4)
Weight at ART initiation (Kg) ^a^	≤43	340 (25)	53	(16)	8.7 (6.6–11.4)	**4 (2.1–7.4)**	**2.5 (1.3–5.1)**
	44–50	389 (29)	42	(11)	5.8 (4.3–7.8)	**2.7 (1.4–5.1)**	**2.2 (1.1–4.4)**
	51–56	285 (21)	12	(4)	2.1 (1.2–3.8)	1	1
	>56	325 (24)	23	(7)	3.8 (2.5–5.7)	1.7 (0.9–3.5)	1.9 (0.9–4)
CD4 count at ART initiation ^a^	≤50	210 (16)	39	(19)	10.5 (7.7–14.4)	**4 (2.2–7.4)**	**2.4 (1.3–4.6)**
	51–200	383 (29)	41	(11)	5.6 (4.1–7.6)	**2.2 (1.2–4)**	1.7 (0.9–3.2)
	201–350	271 (20)	14	(5)	2.5 (1.5–4.3)	1	1
	351–500	227 (17)	13	(6)	3 (1.7–5.2)	1.1 (0.5–2.4)	1.2 (0.5–2.5)
	>500	153 (11)	11	(7)	4 (2.2–7.2)	1.5 (0.7–3.3)	1.5 (0.7–3.4)
	Unknown	95 (7)	12	(13)	8.4 (4.8–14.9)	–	
ART regimen	TDF-based	1,294(97)	123	(10)	5.1 (4.3–6.1)	1	1
	Others	45 (3)	7	(16)	8.8 (4.2–18.4)	1.7 (0.8–3.7)	2 (0.9–4.7)

*LTFU, lost-to-follow-up; PLHIV, people living with HIV; ART, anti-retroviral therapy; Kg, kilogram; TDF, tenofovir*.

*Bold values indicate the statistically significant (p value < 0.05)*.

* *Row percentage*.

a *These variables were measured at the time of ART initiation*.

**Table 3:** Attrition (LTFU and Death) during each year of follow-up and cumulative incidence of attrition in PLHIV initiated on ART between 2015 and 2016 at ART satellite sites, Myanmar.

**Time interval in years after putting on ART**	**Number of PLHIV at start of each time interval**	**lost or dead during each time interval**	**PLHIV censored at the end of each time interval**	**Cumulative incidence of attrition (95% CI)**
0–1	1,339	96	55	7.3 (6–8.9)
1–2	1,188	27	541	10.1 (8.5–11.9)
2–3	620	7	559	11.9 (9.9–14.2)
3–4	54	0	54	11.9 (9.9–14.2)

*LTFU, lost-to-follow-up; PLHIV, people living with HIV; ART, anti-retroviral therapy; 95%CI, 95% confidence interval*.

## Discussion

This is the first study reporting outcomes of PLHIV starting ART at ART satellite sites in Myanmar and demonstrated high rates of retention in care after more than 3 years of follow-up. Previous cohort studies in Myanmar conducted on PLHIV treated at ART centers reported that higher rates of unfavorable outcomes with LTFU ranging from 6.5 to 19% for LTFU and death 9–10% (7–11). There is only a single cohort study published from PLHIV in NAP ART centers which showed a retention in care of 79% over the period 2013 to 2016 (14).

We observed three important findings. First, despite the higher rates of retention in care, most of the attrition happened during the first year after ART initiation. This finding is consistent with the studies reported previously in Myanmar and in other settings (7, 9–11). Attrition was also found to be significantly associated with the expected risk factors, a CD4 count ≤ 50 cells/mm^3^ and having baseline weight ≤ 50 kg. Weight indirectly reflects nutritional status and under 50 kg is considered low for the adult population in Myanmar. Both variables reflect more advanced HIV disease and immune suppression which are risk factors for deaths among HIV patients.

Second, most of PLHIV in this cohort reported heterosexual for HIV transmission risk. As the primary purpose of ART satellite sites is to gain access to hard-to-reach key populations, this finding was not in line with the one of the key objectives of this service delivery model. The Yangon HIV epidemic is expected to include KPs. This could reflect incorrect recording of KP status by health workers or underreporting by patients due to stigma.

Third, this study found that the time taken from enrolment to ART initiation was nearly 2 months despite the national guideline recommended initiating ART as soon as possible among those with CD4 count ≤ 500 cells/mm3, which comprised up to 80% of this cohort. This could reflect delay due to referral and transport time to be seen at ART centres for assessment and initiation and/or time needed for pre-treatment counseling and assessment. It would be important to explore the reasons for these delay (patient, provider, and health system factors) using qualitative research methods as the NAP plans to move to a test and treat strategy. A recently published systematic review reported reasons for delay initiating of ART included both demand-side factors, and supply-side factors (15).

Our study has several strengths. We reported a large cohort of PLHIV on ART receiving care at ART satellite sites, which is the first of this nature in Myanmar. The study used routinely collected data and we have observed very few missing data in our cohort. There are several limitations of this study. First, we only reported data from four out of the available 20 such ART satellite sites in Myanmar and from a single NGO provider, the findings have limited generalizability and should be carefully interpreted. Data from other NGO providers was not routinely available to be provided to the NAP to include in this analysis, reflecting a weakness in the recording and reporting systems that need to be addressed. Second, information on important key clinical variables such as presence of opportunistic infections, CPT, IPT, and HIV viral load, that could be associated with unfavorable outcome were not available. We note data on “migrant” status was not collected, which is an important group in urban Yangon and could have been associated with lower retention in care. Third, as our cohort started at the time of ART initiation, we did not have data to report on the cascade of care from the time of diagnosis to the suppression of viral load. This also may lead to bias toward favorable outcomes as our cohort does not include those enrolled on ART, only those initiated.

Based on the findings, there are some useful programmatic implications. The retention rate after 3 years in this cohort was high, supporting continuation of this model and potential expansion to other remote and hard-to-reach areas with ongoing monitoring and evaluation. Two systematic reviews of peer interventions for HIV have shown an effect on behavioral outcomes but mixed results on clinical outcomes, noting a lack of well-designed comparative studies (16, 17). A study from an Africa country with a similar community based care service model reported high retention among HIV patients on ART compared with the standard of care (73 vs. 44%) (18).

Second, there should be a focus on enhancing data collection systems in ART satellite sites and careful counseling and systematic history taking of HIV transmission risk in order to capture more accurate information to inform program planning. The NAP is currently rolling out an electronic medical record system for HIV and will need to engage NGO providers in its utilization. Efforts should be made to strengthen access to earlier diagnosis given the advanced immune suppression of this cohort. Operational research to describe the HIV care cascade from screening (via the peer outreach model) through to retention in care should be conducted. Assessment of nutritional status of PLHIV at ART satellite sites would be helpful as nutritional interventions could be developed and implemented along with the standard package of care provided at the ART satellite sites. Further quantitative as well as qualitative research could be also undertaken to assess the outcomes at remain ART satellite sites and explore the delays in ART initiation after enrolment.

## Conclusion

We report high rates of retention in care of PLHIV in a new model of ART satellite ART sties in Yangon, Myanmar after 3 years of follow-up. This initial study shows improved outcomes over centralized models in Myanmar (14) and supports continuation of plans to scale-up decentralized community-based care to key populations through ART satellite sites. To optimize outcomes for patients and the program and accelerate progress to reduce HIV transmission, operational research needs to be embedded within the response. Though we can report involvement of peers in HIV care is the essential component which contributed to the success of this model, further factors still needs to be understood, and explored. Further qualitative study is suggested to conduct as it can elaborate the challenges in implementing this model.

## Author Contributions

KH, KS, and MO: study design and protocol development, data collection, data analysis, manuscript writing, approval of final manuscript. SH and HO: study design and protocol development, approval of final manuscript. SM: study design and protocol development, data analysis, manuscript writing, approval of final manuscript.

### Conflict of Interest Statement

The authors declare that the research was conducted in the absence of any commercial or financial relationships that could be construed as a potential conflict of interest.

## References

[1] UNAIDS UNAIDS Data 2017 (2017). Geneva, Switzerland.

[2] UNAIDS UNAIDS. HIV/AIDS Fact Sheet 2016. (2016). Available online at: http://www.unaids.org/sites/default/files/media_asset/UNAIDS_FactSheet_en.pdf (accessed July 8, 2018).

[3] National AIDS Prgoramme National Strategic Plan III for HIV/AIDS in Myanmar (2017). Nay Pyi Taw, Myanmar.

[4] National AIDS Programme; Department of Public Health; Ministry of Health and Sports Myanmar Narrative Report 2015. Naypyitaw (2015).

[5] Suthar AB Rutherford GW Horvath T Doherty MC Negussie EK. Improving antiretroviral therapy scale-up and effectiveness through service integration and decentralization. Aids. (2014) 28 (Suppl. 2) S175–85. 10.1097/QAD.0000000000000259 24849478

[6] Kredo T Ford N Adeniyi FB Garner P Decentralising HIV treatment in lower- and middle-income countries. Cochrane Database Syst Rev. (2013) 6:1–76. 10.1002/14651858.CD009987.pub2 PMC10009870 23807693

[7] Thida A Tun STT Zaw SKK Lover AA Cavailler P Chunn J. Retention and risk factors for attrition in a large public health ART program in myanmar: a retrospective cohort analysis. PLoS ONE. (2014) 9:e108615. 10.1371/journal.pone.0108615 25268903 PMC4182661

[8] Minn AC Kyaw NTT Aung TK Mon OM Htun T Oo MM. Attrition among HIV positive children enrolled under integrated HIV care programme in Myanmar: 12 years cohort analysis. Glob Health Action. (2018) 11:1510593. 10.1080/16549716.2018.1510593 30191749 PMC6136349

[9] Oo MM Gupta V Aung TK Kyaw NTT Oo HN Kumar AM. Alarming attrition rates among HIV-infected individuals in pre-antiretroviral therapy care in Myanmar, 2011-2014. Glob Health Action. (2016) 9:31280. 10.3402/gha.v9.31280 27562473 PMC4999509

[10] Sabapathy K Ford N Chan KN Kyaw MK Elema R Smithuis F. Treatment outcomes from the largest antiretroviral treatment program in Myanmar (Burma): a cohort analysis of retention after scale-up. J Acquir Immune Defic Syndr. (2012) 60:e53–62. 10.1097/QAI.0b013e31824d5689 22334069

[11] Kaung Nyunt KK Han WW Satyanarayana S Isaakidis P Hone S Khaing AA Factors associated with death and loss to follow-up in children on antiretroviral care in Mingalardon Specialist Hospital, Myanmar, 2006-2016. PLoS ONE. (2018) 13:e0195435 10.1371/journal.pone.0195435 29621302 PMC5886568

[12] Department of Population Ministry of Immigration and Population The 2014 Myanmar Population and Housing Census The Union Report. Vol. 2 (2015).

[13] National AIDS Programme National AIDS Programme, Ministry of Health and Sports, Annual Report (2016).

[14] Aung ZZ Oo MM Tripathy JP Kyaw NTT Hone S Oo HN. Are death and loss to follow-up still high in people living with HIV on ART after national scale-up and earlier treatment initiation? A large cohort study in government hospital-based setting, Myanmar: 2013-2016. PLoS ONE. (2018) 13:e0204550. 10.1371/journal.pone.0204550 30252904 PMC6155560

[15] Ahmed S Autrey J Katz IT Fox MP Rosen S Onoya D Why do people living with HIV not initiate treatment? A systematic review of qualitative evidence from low- and middle-income countries. Soc Sci Med. (2018) 213:72–84. 10.1016/j.socscimed.2018.05.048 30059900 PMC6813776

[16] Medley A Kennedy C O'Reilly K Sweat M. Effectiveness of peer education interventions for HIV prevention in developing countries: a systematic review and meta-analysis. AIDS Educ Prev. (2009) 21:181–206. 10.1521/aeap.2009.21.3.181 19519235 PMC3927325

[17] Simoni JM Nelson KM Franks JC Yard SS Lehavot K. Are peer interventions for HIV efficacious? A systematic review. AIDS Behav. (2011) 15:1589–95. 10.1007/s10461-011-9963-5 21598034 PMC3607378

[18] Kelly JD Frankfurter R Lurton G Conteh S Empson SF Daboh F. Evaluation of a community-based ART programme after tapering home visits in rural Sierra Leone: a 24-month retrospective study. SAHARA J. (2018) 15:138–45. 10.1080/17290376.2018.1527244 30257611 PMC6161614

