# Supplementary material for: Early Success With Retention in Care Among People Living With HIV at Decentralized ART Satellite Sites in Yangon, Myanmar, 2015–2016
Source: Front Public Health. 2019 May 22;7:124. doi: 10.3389/fpubh.2019.00124 (PMC6538688; doi:10.3389/fpubh.2019.00124)
Supplement: Supplementary file 1 [file Data_Sheet_1.docx]

Annex 1

Lists of ART satellite sites in Myanmar

| Organization | ART satellite sites |
| --- | --- |
| PSI (22 sites) | 1. Bago |
|  | 1. Hinthada |
|  | 1. Lashio |
|  | 1. Chan Aye TharZan |
|  | 1. PyiKyi Ta Khon |
|  | 1. Thabeikkyin |
|  | 1. Singu |
|  | 1. Mawlamyine |
|  | 1. Meikhtila |
|  | 1. Mogoke |
|  | 1. Monywa |
|  | 1. Myeik |
|  | 1. Myingyan |
|  | 1. Myitkyina |
|  | 1. Nyaung-U |
|  | 1. Pathein |
|  | 1. Pyay |
|  | 1. Pyinoolwin |
|  | 1. Sittwe |
|  | 1. Taunggyi |
|  | 1. Taungoo |
|  | 1. Yangon |
| MAM (5 sites)  (Study population in this study) | 1. HlaingTharyar |
|  | 1. ShawePyiThar |
|  | 1. Thanlyin |
|  | 1. Thanphyuzayat |
|  | 1. Puta O |
| Alliance (1 site) | 1. Yangon |

Annex 2

Lists of abbreviations

HIV: Human Immunodeficiency virus

ART: Anti-retro viral therapy

AIDS: Acquired Immune Deficiency Syndrome

PLHIV: People Living with HIV

KPs: Key Populations

PWID: People who inject drugs

MSM: Men who have sex with men

FSW: Female sex workers

UNAIDS: The Joint United Nations Programme on HIV/AIDS

NAP: National aids Programme

iNGOs: International non-governmental organizations

NGOs: Non-governmental organizations

NSP: National Strategic Plan

WHO: World Health Organization

DC: Decentralized

LTFU: Lost to follow-up

HTS: HIV testing services

VCCT: Voluntary Confidential Counselling and Testing

GFATM: the Global Fund against AIDS, TB and Malaria

HR: Hazard rtios

CD4 count: It is a test that measures how many CD4 cells in the blood. The CD4 count should increase in response to effective ART.
